# Polymer Fibers Covered by Soft Multilayered Films for Sensing Applications in Composite Materials

**DOI:** 10.3390/s19184052

**Published:** 2019-09-19

**Authors:** Dorian Nikoniuk, Karolina Bednarska, Maksymilian Sienkiewicz, Grzegorz Krzesiński, Mateusz Olszyna, Lars Dähne, Tomasz R. Woliński, Piotr Lesiak

**Affiliations:** 1 Faculty of Physics, Warsaw University of Technology, 00-662 Warsaw, Poland; d.nikoniuk@gmail.com (D.N.); karolina.bednarska.dokt@pw.edu.pl (K.B.); tomasz.wolinski@pw.edu.pl (T.R.W.); 2 Faculty of Power and Aeronautical Engineering, Warsaw University of Technology, 00-665 Warsaw, Poland; sienkiewicz.maksymilian@gmail.com (M.S.); gkrzesin@meil.pw.edu.pl (G.K.); 3 Surflay Nanotec GmbH, 12489 Berlin, Germany; m.olszyna@surflay.com (M.O.); l.daehne@surflay.com (L.D.)

**Keywords:** fiber optic sensors, polymer optical fibers, photonic crystal fibers, composite materials, embedded sensors, layer-by-layer self-assembly

## Abstract

This paper presents the possibility of applying a soft polymer coating by means of a layer-by-layer (LbL) technique to highly birefringent polymer optical fibers designed for laminating in composite materials. In contrast to optical fibers made of pure silica glass, polymer optical fibers are manufactured without a soft polymer coating. In typical sensor applications, the absence of a buffer coating is an advantage. However, highly birefringent polymer optical fibers laminated in a composite material are much more sensitive to temperature changes than polymer optical fibers in a free space as a result of the thermal expansion of the composite material. To prevent this, we have covered highly birefringent polymer optical fibers with a soft polymer coating of different thickness and measured the temperature sensitivity of each solution. The results obtained show that the undesired temperature sensitivity of the laminated optical fiber decreases as the thickness of the coating layer increases.

## 1. Introduction

Traditional metallic alloy parts can be replaced with great success by composite materials. Their high strength, low weight, and cost effectiveness make them ideal candidates for manufacturing parts to be used in aviation, marine, and windmill industries [1,2,3].

Mechanical properties of polymer composites critically depend on the lamination process. Consequently, there is a strong need for the monitoring of polymer matrix curing in order to ensure that it has been performed according to predefined requirements. The lamination process strongly influences the final properties of a polymer composite, for example its glass transition temperature or ultimate tensile strength. An ideal solution is in vivo monitoring of the lamination process to enable the introduction of instantaneous corrections. This would be possible if, for instance, fiber optic sensors are embedded directly in a composite material. However, fiber optic sensors are highly sensitive to transverse deformations that arise during the production process of a composite material [4].

Polymerization is a chemical process that consists of the simple joining of multiple molecules containing multiple bonds into a single macromolecular compound without any byproducts [5,6]. This process can be initiated under the influence of visible light activation, chemical reaction, or temperature. During polymer formation, we encounter the problem of polymerization shrinkage, which consists of bringing the molecules closely together due to the Van der Waals forces.

**Figure 1:**
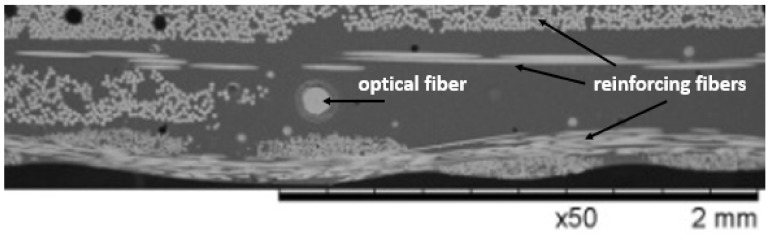
A typical arrangement of the optical fiber in a composite material.

In the case of fiber optic sensors embedded in polymer composites, the situation is complex. The displacement vectors are directed centrally to the lateral surface of the optical fiber (symmetrically in the fiber cross section, similarly to the hydrostatic pressure) only when the reinforcement fibers are arranged parallel to the optical fiber axis. However, if the reinforcement fibers form a two-dimensional braided fabric (Figure 1), then the displacement vectors are asymmetrical, and mainly directed to the surface of the layer. The optical fiber placed in the space between the two fabrics is then compressed. The positioning of these fibers has a decisive effect on the type of deformation to which the laminated optical fibers are exposed. The optical fiber placed in the space between two fabrics is then subjected mainly to compressive stresses.

During the lamination process, deformations in the laminated fibers arise due to polymerization shrinkage. Such material contraction may, under the least favorable circumstances, completely change the characteristics of the fiber optic sensor placed in it. A solution to the problem was used in the case of optical fibers made of silica glass soft coatings which isolate the sensor from the transverse stresses occurring in the material [7]. In our previous work [8], we studied the influence of thermal expansion of composite materials on embedded polarimetric sensors. We found that a polarimetric fiber optic sensor laminated in a composite material without any soft buffer coating was characterized by greater temperature sensitivity than a sensor laminated with a soft buffer coating. Numerical calculations presented in [9] show that a soft protective coating is able to absorb both stresses generated during the lamination process and stresses created by the thermal expansion of a composite material.

**Figure 2:**
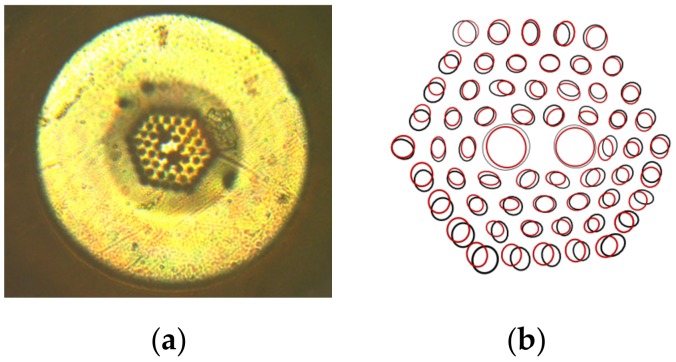
A picture of laminated microstructured polymer optical fiber (mPOF) manufactured by Kiriama Pty Ltd. of Sydney, Australia (**a**) and a comparison between cross sections of polymer photonic crystal fibers before (black ellipses) and after (red ellipses) the lamination process (**b**) [9].

However, it may also lead to deterioration of sensor parameters, as the optical fiber will not register longitudinal deformation correctly, and will not function properly, due to the mismatch of Young’s modulus between silica glass and soft polymer. The solution to this problem may be the use of an optical fiber made of polymethyl methacrylate (PMMA) [10,11], thermoplastic cyclic olefin copolymer (TOPAS^®^) [12], polycarbonate (PC) [13], or ZEONEX [14], whose Young’s moduli are much better matched to the composite material [15]. PMMA is one of the most widely used optical polymers for polymer optical fiber sensors [16]. In the case of lamination in composite materials, it is necessary to apply an additional layer of soft coating which can effectively protect the optical fiber from external stress. However, the standard polymer, photonic crystal fiber, has no acrylate coating and is very sensitive to the stresses occurring in the composite material during temperature changes. The deformations presented elsewhere [9] (Figure 2) indicate that small holes in the laminated fiber were compressed towards to the fiber core. Additionally, the ellipticity of the core was reduced and the diameter of the two big holes near the core decreased. These deformations of the structure were different along both the x and y axes, since there is such a visible change in fiber geometry. Comparing the sensitivity to the temperature of microstructured polymer optical fibers (mPOF) before and after the lamination process [17], it should be noted that polymer optical fibers increased their thermal sensitivity in a significant way. The reason for this is the polymerization shrinkage, which in an uncontrolled way changes the birefringence of the mPOF laminate in polymer composites with a two-dimensional structure created by the reinforcing fibers. During polymerization, this structure may strengthen or weaken the birefringence in laminated mPOF and, consequently, change the sensitivity of this optical fiber to any deformation.

Soft polymer coating can be performed by using a layer-by-layer (LbL) self-assembly process involving electrostatic interaction. This almost 30-year-old deposition technique is an approach toward a simple, controllable, and low cost fabrication of nanoscale films connected with functionalization of planar or spherical surfaces. The basic principle is an alternating adsorption of charged materials (polyelectrolytes, nanoparticles, etc.) onto an oppositely charged surface [18]. The most physicochemical surface properties, such as thickness, elasticity, permeability, or reactivity, can be controlled by the selection of polymers and modification of deposition parameters (ionic strength, pH value, etc.). As reported in [19], the use of natural biopolymers (chitosan, alginate, hyaluronic acid, etc.) in a multilayer build up leads to the formation of strongly hydrated and thick LbL films, giving better protection from external mechanical stress factors. However, a thick multilayer film can also reduce the sensing magnitude of an optical fiber embedded in a composite material, making them completely useless. Hence, precise thickness control and selection of suitable polymers for optimal soft multilayered film are required.

The LbL process is a self-limiting deposition process. This means that if this process is carried out under the same conditions each time (with the same ion strength and pH after a defined time of deposition, and for >15 min), it always yields the same and very reproducible layer thickness, of nanometer accuracy on each point, independent of the structure of the object or the concentration of the deposition material. Other methods, like chemical reaction on the surface, yield either an extremely thin monomolecular layer, or, in the case of a chain reaction such as radical polymerization, a hardly controllable thickness. Other methods, like dipping in a polymer solution or chemical vapor deposition, are also difficult to control or inhomogeneous on the surface. Therefore, the LbL method is very versatile and suitable for the homogeneous and controlled deposition of a number of chemical compounds.

In this paper, the possibility of using mPOF with a soft polymer film for monitoring the degradation of composite materials has been presented. In traditional applications, there is no need for the mPOF to be covered with an additional layer of soft polymer, therefore during the lamination process they are exposed to additional stresses that affect the mPOF parameters. In order to protect optical fibers from polymerization shrinkage, we present the possibility of coating them with a soft polymer film by using the LbL technology and show that protected mPOFs retain their properties after the lamination process.

## 2. Materials and Methods

In this research, we used a polymer polarization maintaining (PM) photonic crystal fiber manufactured by Kiriama Pty Ltd. of Sydney, Australia. This mPOF is characterized by a 2.5 × 4.5 μm core diameter, 4.4 μm hole spacing, and 2.2 μm and 4.5 μm small and big hole diameters, respectively. The core and cladding were made of PMMA, and were surrounded by a PC coating.

### 2.1. Sample Fabrication

The soft polymer coating on the mPOFs was processed with thicknesses (δ) of 100, 300, and 500 nm using positively charged polysaccharide-chitosan (CHI) of low molecular weight (Chitopharm-S, MW 50–100 kDa, Cognis, Monheim am Rhein, Germany), and negatively charged synthetic polymer-poly(styrene sulphonate) (PSS) (MW 70 kDa, Sigma Aldrich, St. Louis, MI, USA). A multilayer film was deposited directly onto the mPOFs at a pH of 5.6 (50 mM sodium acetate buffer) in the presence of 200 mM NaCl by alternately immersing the substrate in polyelectrolyte solutions for 20 min. After the deposition of each layer, the mPOFs were washed three times in deionized water. Finally, the LbL-coated mPOFs were dried under a nitrogen stream.

In principle, the thickness of the dry film could be measured by neutron scattering or electron microscopy. However, the first method was not available, and the second requires an exact cutting procedure of the fiber. Hence, the thickness of the CHI/PSS film was calculated using the values obtained by Hatami et al. in [20].

The composite plate comprised eight fiberglass layers: two outer layers made of Interglass 92–100, and six inner layers made of Krosno STR-450. Two PM mPOFs were implemented into the composite materials between the outer and inner layers. The dimensions of the sample were 255 × 42 × 2.5 mm. Both types of samples were prepared at the Faculty of Materials Science and Engineering, Warsaw University of Technology.

A thermally conductive aluminum plate was used to make the heating area of three Peltier modules uniform. The dimensions of the plate were 255 × 42 × 1 mm. The outer layer of the sample was in contact with the plate.

### 2.2. Characterization Methods

Changes in polarization induced by temperature are measured with a polarimeter. A polarization controller is used to set the state of polarization (SOP) [21,22] at the input to the polarimetric sensors. This is done by changing the azimuth and ellipticity of the polarization at the controller. The initial values are set in such a way that a maximum optical power is achieved at the input of the polarimeter.

The state of polarization at any time can be represented by three normalized Stokes vector parameters. The Poincaré sphere is a tool used to graphically represent the transformations of the polarization, where the Stokes parameters define a point on the sphere in a Cartesian, right-handed coordinate system. The corresponding states of polarization are assigned to areas on the sphere. In particular, longitudes represent the states of the same azimuth, while latitudes represent the same ellipticity. Because any SOP is represented by a single point on the sphere, a continuous path on the surface of the Poincaré sphere represents the continuous changes in the azimuth and ellipticity of the polarization. If the path of the point is a full circle, then the phase shift is 2π. Hence, by evaluating the data obtained from the Poincaré sphere, both the strain and temperature responses of a polarimetric sensor can be acquired.

**Figure 3:**
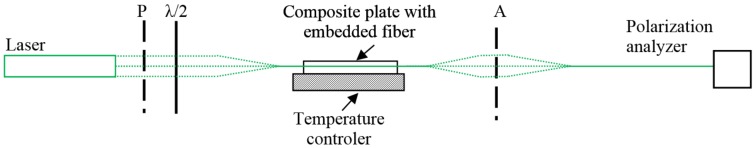
Measurement setup in which P and A are the polarizer and analyzer, respectively.

The experimental setup is shown in Figure 3. A 543 nm laser was used as an input source and was launched with its polarization at a 45° angle with respect to the optical axis of the PM fiber, so that the optical intensities of the two orthogonal polarizations were evenly distributed. The input angle was controlled by using a polarizer and a half wave plate. A Thorlabs TXP 5004 polarimeter was used to measure the temperature effects of the mPOF. A polarimeter provided information about the SOP of the mPOF output, presented graphically as a trace on the Poincaré sphere. The temperature in the experiment was changed continuously, whereas the induced phase shift for an applied temperature change was calculated from the Poincaré sphere.

## 3. Results

### 3.1. Numerical Calculations

The finite element method (FEM) is a commonly used tool for calculating equations and sets of partial differential equations describing various aspects of mathematical physics. A solution is obtained as a result of discretization of given area and boundary (initial) conditions, and defining degrees of freedom (usually these are values of the sought function in the nodes of the discretized net, e.g., components of the displacement vector, temperature, or electric field potential). The discretization of the analyzed area consists of dividing into subareas, called finite elements, connected by nodes.

Nowadays, calculation packages allow advanced phenomena (static, dynamic, linear, or nonlinear analyses) to be modeled, as well as work with complex constructions or materials (plating of aircrafts, layered, or anisotropic materials). For the analysis presented further in this manuscript, we used ANSYS v19.0 software.

**Figure 4:**
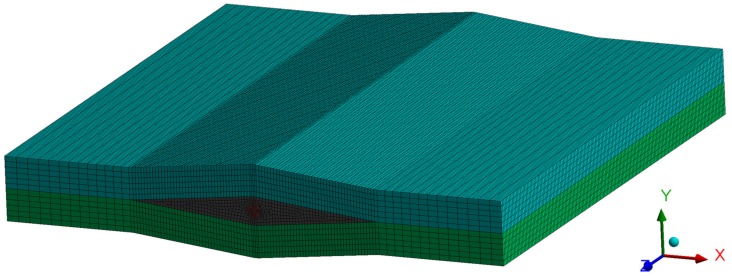
The finite element method (FEM) model used in analysis.

The aim of the conducted simulations was to calculate the influence of polymer coating thickness on the distribution of stresses in the fiber. Their occurrence is connected to the technological shrinkage of a composite, which appears during the manufacturing process. The coating’s task is to level the effects of the appearing stresses. For this analysis, we created the model of a fragment of a composite with an embedded fiber. Four configurations of δ were analyzed: 0 nm (no coating), 100 nm, 300 nm, and 500 nm. The FEM model is presented in Figure 4.

Owing to the local influence of the coating on the distribution of stress, the composite model was limited to two layers. The fiber was placed between those layers. We also included the local deformation of the composite due to the embedding of a fiber sensor in the models. The void created in this way was filled with a resin.

**Figure 5:**
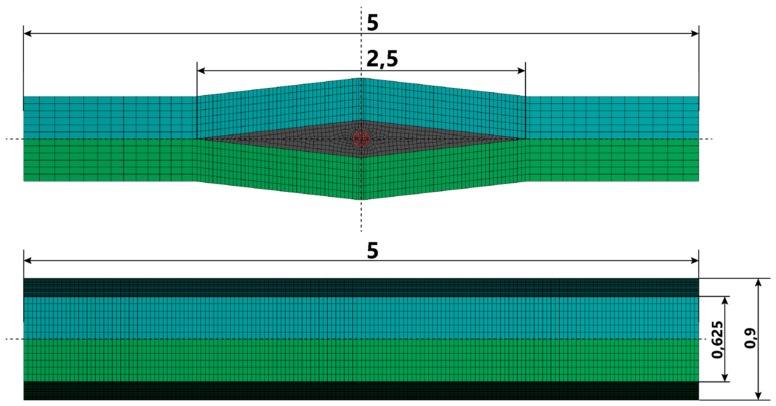
Geometry of the models.

**Figure 6:**
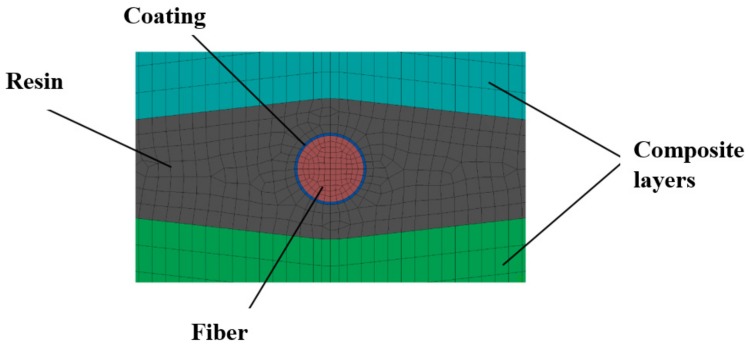
The scheme of all elements used in the analysis.

**Figure 7:**
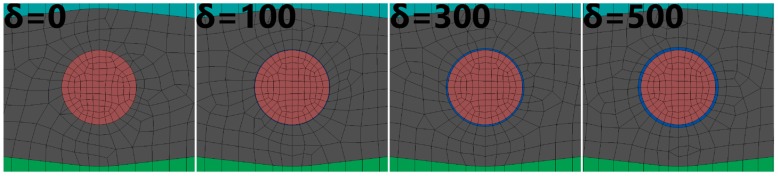
Zoom on the embedded fiber. All analyzed cases are presented.

Figure 5, Figure 6 and Figure 7 present the geometry of the FEM models, along with the description of individual elements. The diameter of the fiber was r = 125 µm.

The linear shrinkage of the resin during the cooling process was carried out by thermal expansion of the material. The value of the thermal expansion coefficient for the resin was set at 0.00001 1/°C, while for the other materials it was equal to 0. Thus, if a decrease in temperature by 100 °C is assumed, the resin is subjected to shrinkage by 1‰. The remaining elements (layers of the composite, coating, and fiber) were not deformed due to a change in temperature, but only due to mutual influences. Supporting conditions were included so as to preserve the freedom and symmetry of the model’s deformation.

**Figure 8:**
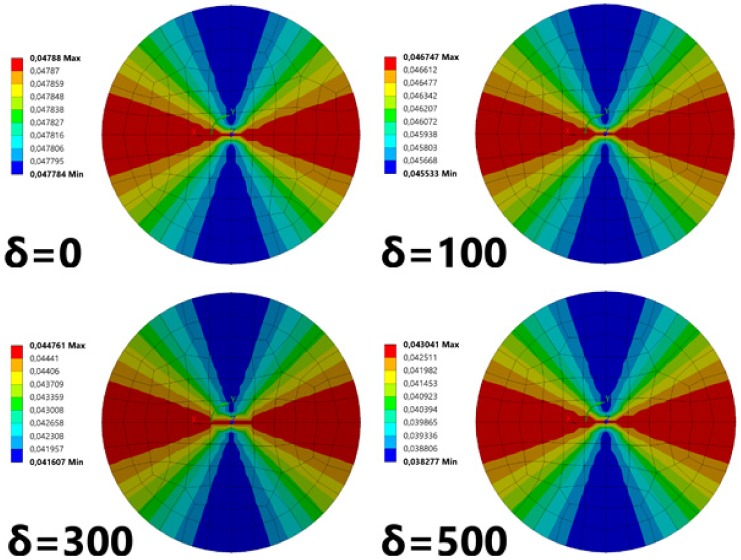
Radial stresses σ obtained in the cross section of the fiber for all cases of the coating thickness δ. Units are in MPa. The increasing layer thickness causes a decrease in the radial stress.

Due to the qualitative characteristics of the analysis, we decided to use the materials available in the library of ANSYS software. For the composite material, we chose a composite fabric made of carbon fiber. Its mechanical properties are presented in Table 1, whereas the mechanical properties of the remaining materials used in the analysis are presented in Table 2. The definitions of the given parameters are: E, Young’s modulus; ν, Poisson’s ratio; G, shear stiffness; and α, expansion coefficient. Figure 8 presents the radial stresses in the cylindrical coordinate system connected with the fiber.

**Figure 9:**
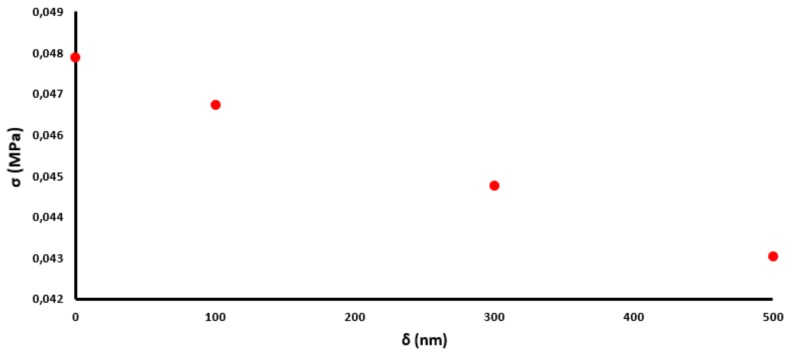
Radial stresses inside the fiber as a function of the coating thickness.

The results of the conducted analyses indicate that radial stresses decrease with an increasing thickness of the coating. This change is nearly linear, which can be observed in Figure 9.

The numerical calculation shows that the distribution of stresses is nonuniform, but both maxima and minima are not significantly different (for a 100 nm coating, the values change from 0.0455 MPa to 0.0467 MPa). This means that the shape of the cross section, which was initially circular, is slightly deformed into an ellipse (Figure 2).

**Figure 10:**
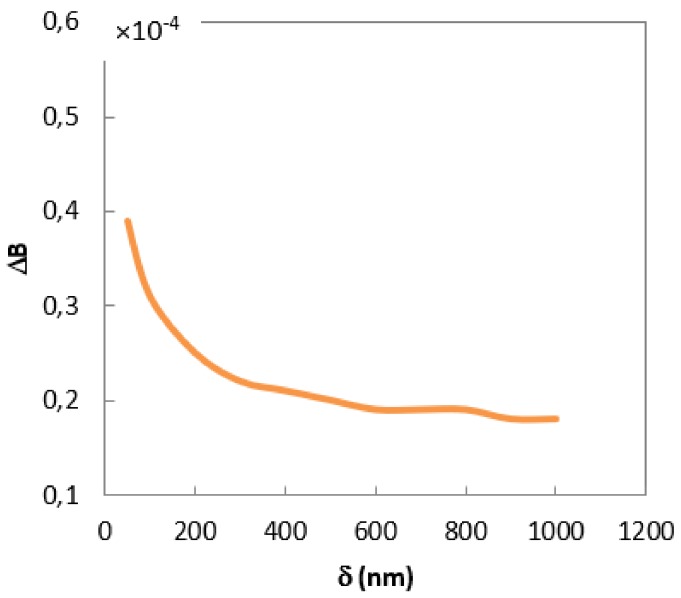
Numerical calculations of the birefringence change of an mPOF under the influence of a 1‰ polymerization shrinkage for soft coatings of different thickness.

To calculate the two-dimensional stress distribution in the fiber, and the effect of strain on light propagation, we used the values obtained in ANSYS analysis for simulations in COMSOL Multiphysics^®^ software (Comsol Ltd., Stockholm, Sweden), which is also based on the finite element method. The use of the COMSOL software allowed us to calculate the point force and propagation constants of the two confined modes under the influence of hydrostatic pressure. A comprehensive description of this procedure can be found in our previous work [9]. A detailed numerical analysis shows that the use of a buffer zone of submicrometer thickness allows for a significant reduction of an undesirable impact of external factors on the optical fiber. Figure 10 shows how the birefringence of the mPOF changed under the influence of a 1‰ polymerization shrinkage if the thickness of the CHI/PSS coating was modified. If the thickness δ of the CHI/PSS film (characterized by a Young’s modulus of 3 MPa) was below 500 nm, then a significant decrease in the influence of the polymerization shrinkage on the optical fiber was observed. However, for δ higher than 500 nm, any additional decrease did not occur.

### 3.2. Experimental Results

**Figure 11:**
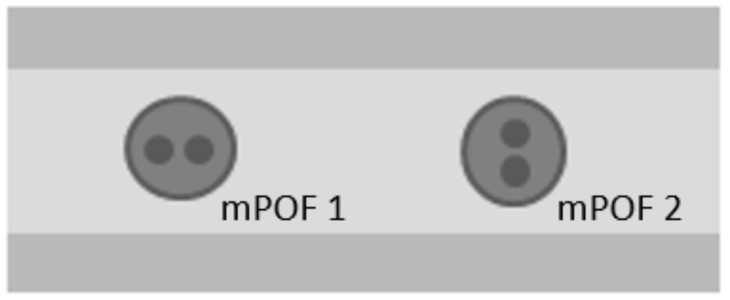
Two orientation types of the mPOF fiber in a composite material.

**Figure 12:**
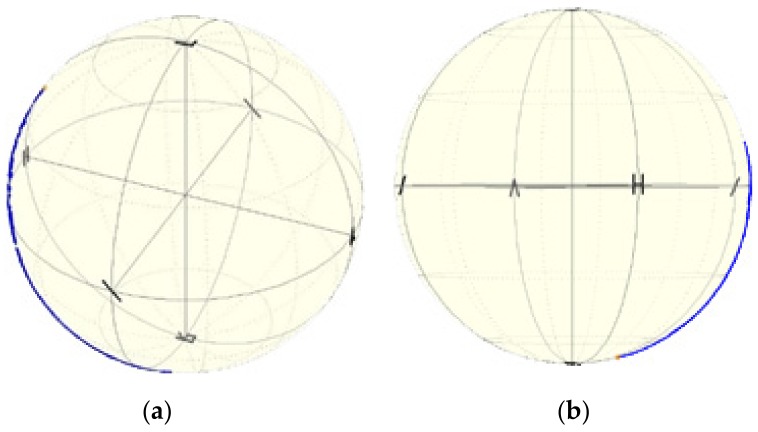
Phase shift observed in laminated mPOF oriented at 0° for ΔT = 1 °C (**a**) and oriented at 90° for ΔT = 5 °C (**b**).

Firstly, the mPOF was placed in the composite material at different angles to the reinforcement layers (0 and 90 ° presented in Figure 11). The observed change of birefringence under the influence of a temperature change (Table 3) was the strongest for optical fiber (a) and the smallest for optical fiber (b) (Figure 12). This is due to the fact that the additional stresses induced during the lamination process strengthened the birefringence of mPOF (a) and compensated for the birefringence of mPOF (b). Both high and low birefringence change the phase of the propagating light under the influence of a temperature change more or less adequately. In order to measure the effect of soft buffer coating, all optical fibers should be placed at the same angle with respect to the reinforcement layers.

**Figure 13:**
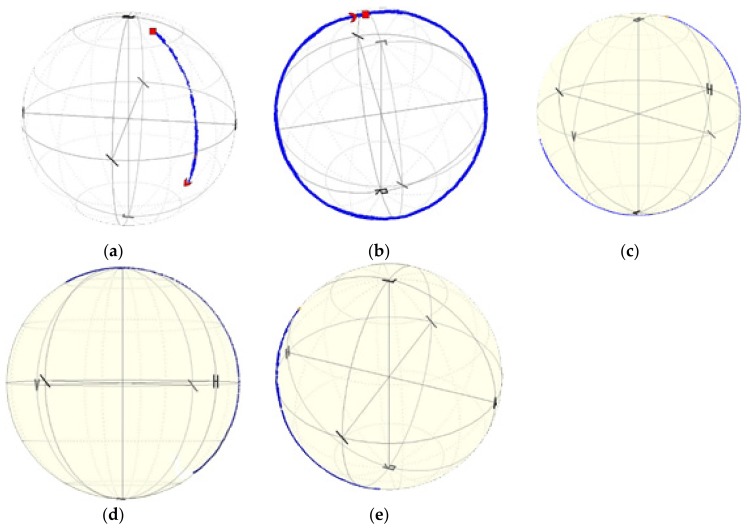
Observed phase shift in mPOF for a temperature change from 21 °C to 24 °C in free space (**a**) and phase shift in mPOF in a composite material for the same temperature variations with no coating (**b**), with a 100 nm coating (**c**), with a 300 nm coating (**d**), and with a 500 nm coating (**e**).

**Figure 14:**
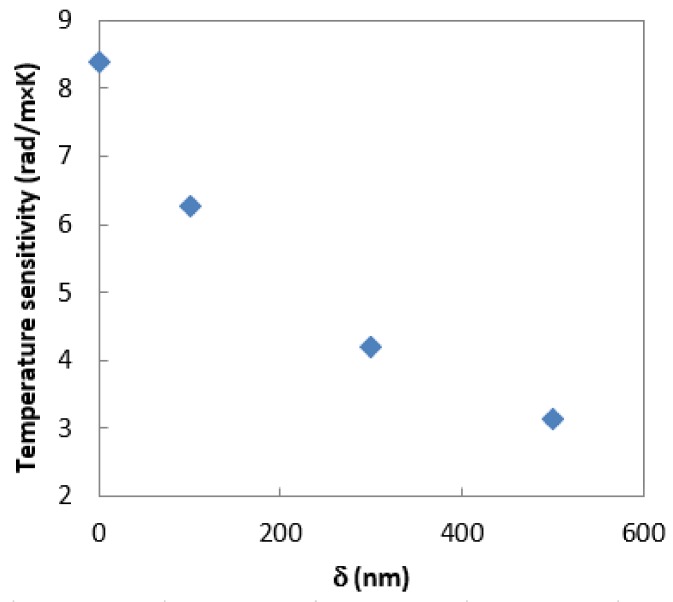
Measured temperature sensitivity of the laminated mPOF as a function of the coating thickness.

Figure 13 presents the experimental validation of the described numerical calculations. The phase shift observed for uncoated fiber resulting from an increase in temperature around the laminated fiber by 3 °C is presented on the Poincaré sphere as a full circle. When the thickness of the CHI/PSS coating increases, the temperature sensitivity of the laminated photonic polymer optical fiber decreases. The obtained results show that at 100 nm of the coating thickness, the phase shift for the temperature change around the laminated fiber by 3 °C is presented on the Poincaré sphere as ¾ of the full ring, and the calculated temperature sensitivity of the laminated mPOF is equal to 6.28 $\left(rad/m\times K\right).$ Further increasing δ to 300 nm and 500 nm leads to a decrease in temperature sensitivity to 4.19 $\left(rad/m\times K\right)$ and 3.14 $\left(rad/m\times K\right)$, respectively. The value observed at a coating thickness of 500 nm (Table 3) is close to the temperature sensitivity stored in a free space. This result means that the birefringence of the optical fiber changed to a negligible extent during the lamination process. Additionally, the coating layer adequately protects the laminated fiber against the deformations of the composite material resulting from thermal changes. The character of calculated stress changes (Figure 9) and birefringence (Figure 10), as well the measured temperature sensitivity (Figure 14), show very high compatibility. In all cases, increasing δ above 500 nm shows a decrease in the dynamics of the effect of polymerization shrinkage on the mPOF.

## 4. Conclusions

In this paper, the influence of temperature on the stress sensitivity of mPOF placed in the composite material has been investigated and analyzed. As the ANSYS analysis showed, the mPOF was distorted, which has been confirmed by observations of the cross section of the laminated optical fiber. Such a deformation has a very negative effect on the birefringence of the laminated fiber, and on its sensitivity to temperature changes. The results presented indicate that the LbL coating consisting of CHI/PSS multilayers reduces both the deformations of a polymer fiber optic during the lamination process, and the thermal changes in a composite material. This holds great potential for enhanced functionalities of PM mPOFs, as well as their use in control of manufacturing processes, while simultaneously improving their practical applications in many industrial fields, especially in structural health monitoring.

## Figures and Tables

**Table 1:** Mechanical properties of the composite.

E11(MPa)	E22 (MPa)	E33 (MPa)	ν12	ν23	ν13	G12 (MPa)	G23 (MPa)	G13 (MPa)	α (1/°C)
61340	61340	6900	0.04	0.3	0.3	19500	2700	2700	0

**Table 2:** Mechanical properties of the remaining materials.

	Resin	Fiber	Coating
E (MPa)	10	3000	3
ν	0.35	0.345	0.3
α (1/°C)	0.00001	0	0

**Table 3:** Measured temperature sensitivity of the laminated mPOF $\left(rad/m\times K\right)$.

				Coating		
Free Space	Radial	Horizontal	No Coating	100 nm	300 nm	500 nm
2.09	7.85	1.26	8.38	6.28	4.19	3.14
